# Calculation of Capacitive-Based Sensors of Rotating Shaft Vibration for Fault Diagnostic Systems of Powerful Generators

**DOI:** 10.3390/s22041634

**Published:** 2022-02-19

**Authors:** Ievgen Zaitsev, Victoriia Bereznychenko, Mohit Bajaj, Ibrahim B. M. Taha, Youcef Belkhier, Vladyslav Titko, Salah Kamel

**Affiliations:** 1 Department of Theoretical Electrical Engineering and Diagnostics of Electrical Equipment’s, Institute of Electrodynamics of NAS of Ukraine, Peremohy Str., 56, 03057 Kyiv, Ukraine; vika.bereznichenko@i.ua (V.B.); votitko@ied.org.ua (V.T.); 2 Department of Applied Information Systems, Faculty of Information Technology, Taras Shevchenko Nation University of Kiev, Volodymyrska Str., 64/13, 01601 Kyiv, Ukraine; 3 Department of Electrical and Electronics Engineering, National Institute of Technology Delhi, Delhi 110040, India; mohitbajaj@nitdelhi.ac.in; 4 Department of Electrical Engineering, College of Engineering, Taif University, P.O. Box 11099, Taif 21944, Saudi Arabia; 5 Department of Computer Science, Faculty of Electrical Engineering, Czech Technical University in Prague, Karlovo Namesti 13, 121 35 Praha, Czech Republic; belkhieryoucef@outlook.fr; 6 Department of Electrical Engineering, Faculty of Engineering, Aswan University, Aswan 81542, Egypt; skamel@aswu.edu.eg

**Keywords:** capacitive sensor, fault diagnostic, rotating shaft vibration

## Abstract

This paper presents the results of research and development of capacitive-based sensors of rotating shaft vibration for fault diagnostic systems of powerful turbines and hydro generators. It showed that diagnostic systems with special sensors are the key to increasing the reliability of powerful turbines and hydro generators. The application of sensors in monitoring systems was considered, and the requirements for the sensors used were analyzed. Structures of concentric capacitive-based sensors of rotating shaft vibration based on the measurement of the capacitance value from the distance to the metal surface were proposed. The design scheme was created for determining electrode dimensions of the rotating shaft vibration capacitive-based sensors with concentric electrodes, and analytical dependences were obtained. The calculation results allow the selection of optimal parameters of the active and guard electrodes. Analytical and computer simulation methods determined the response functions of the capacitive sensors. Analytical calculations and simulation results using 3D FEM were used to find the response functions of the sensors. The calculation of the characteristics of the capacitive-based sensors of rotating shaft vibration is presented. The study of the influence of fringe effects was carried out using the obtained results of the modeling and analytical calculations.

## 1. Introduction

Electrical networks are complex, integrated systems, so it is important to ensure their proper interaction with generating companies with an understanding of the electrical characteristics of their equipment and operational dynamics. An important direction in the development of the electricity industry is to ensure the stable, reliable, and safe operation of all energy system elements. The energy system was changed and became intelligent networks built on the concept of the Smart Grid. Rapid development and implementation of intelligent technologies made it possible to develop and implement online monitoring and control of equipment, processes, and energy systems under IEC 63097: Smart Grid Roadmap at all stages, from production, transmission, and distribution to energy consumption.

Applying control and monitoring methods and tools is an effective way to ensure the high reliability and safety of the power system. Obtained information allows distribution and supply system operators to forecast and optimize the operation of the system and power equipment, determine the current technical condition of power grid equipment, and keep track of defects until an accident happens.

Control and monitoring methods and tools in power systems use a feature called the non-standard and sometimes uniqueness of tasks for implementing measurement tools of electrical equipment’s control and diagnostic parameters. For this reason, the control and monitoring of the condition of power plant equipment are carried out under difficult conditions (high voltage, electromagnetic interference, and a wide range of temperatures, vibrations, explosive, and fire-hazardous and corrosive environments) in places with tight space. Vibration diagnostics are widely used to obtain information on the stage of powerful generators, which allows one to determine a significant number of defects in powerful generators [1,2]. These systems are an effective way to develop most control and monitoring tools. Engineers are aware of the hazards and consequences that critical vibration can cause in rotating generators [3,4]. Vibration diagnostics are widely used to obtain information on the state of powerful generators, which allows one to determine a significant number of defects in powerful generators, which lead to vibrations and deformations of structural elements of the machine [5]. One of the key parameters that allow the state of a powerful rotating generator to be determined is rotating shaft vibration or run-out control [6,7,8,9].

Today, some stations have rotating shaft vibration or run-out control systems [10,11,12]. The basis for obtaining information in control systems is that the primary measuring transducers (sensors) of various physical quantities into electrical signals must meet fairly stringent requirements (accuracy, resolution, reproducibility, and stability of characteristics over time). The automatic measurement of vibration in systems is carried out using non-contact eddy current [13,14], capacitive [15], and optical sensors [16].

Capacitive sensors provide high measurement accuracy and long-term stability of characteristics [17]. They can also operate in conditions of strong electromagnetic fields [18,19]; allow fundamentally linear transfer characteristics to be obtained [17]; and are distinguished by high accuracy, stability, and noise immunity to the external environment of the generator, in contrast to eddy current sensors [9].

Capacitive sensors used to diagnose equipment to detect mechanical faults are usually aimed at monitoring changes in parameters in spatial (position, micro displacement, and geometric shape) structural elements of the generator unit. The characteristics of capacitive sensors that measure the dielectric characteristics of materials with different electrodes for non-destructive testing of materials with one-way access to an object with different dielectric constants have been well studied [20,21,22,23,24,25,26,27,28,29]. These works have focused on the results presented by studies that used capacitive sensors to solve problems of control identification defects of widespread materials in industry. Among them are composite materials for aircraft construction [26,28,29,30], polymeric materials [20], moisture sensors for orthotropic (fibers, tapes, paper, veneer, etc.) [21] and human skin materials [24], quality sensors for concrete slabs [25], multilayer dielectrics [27,28,29], and others. All presented analytical models for calculating characteristics of capacitive sensors are based on simplified idealized models. The use of models based on idealized analytical models has been limited by the accuracy of calculating the characteristics of sensors for real designs and can lead to significant errors due to differences between theoretical and experimental data. This can lead to the receipt of false data when assessing the condition of power equipment. Therefore, unfortunately, it is not possible to apply the results of these studies to solving the problem of measuring the displacement of the grounded flat surface of the rotating shaft with respect to the plane of the concentric electrode. Therefore, the design and development of structurally optimal sensors for diagnosing the mechanical parameters of units of energy objects should be carried out using numerous computer simulation tools.

The use of existing capacitive sensors in standard diagnostic systems is limited because of the need to adapt them to the generator control unit, which is not always possible because of the design features of the generators in operation. Therefore, new sensors should be developed, taking into account the design features and operating modes of generators as well as the external working environment of powerful generators (high voltages, powerful magnetic fields of up to 1.5 T, high temperatures, increased vibration, etc.) [18,19].

One of the most critical stages in the development of sensors is the design process. The sensor design process is characterized by significant analytical works and calculations and physical modeling using full-scale prototypes. The sensor development process requires a significant number of analytical calculations and the realization of many physical simulations on full-scale prototypes; this is because of the complexity of energy facilities, especially powerful turbo and hydro generators. The analysis of large number of experimental studies involves a difficult and long process that requires significant financial and human potential.

Therefore, the main goals of this research are the following: to study capacitive-based sensors of rotating shaft vibration with concentric electrodes for fault diagnostic systems of powerful generators; to determine the optimal geometric dimensions of the radius for the sensor electrodes; and to determine the response function and fringe effect error versus the geometric dimensions of the sensor electrode.

## 2. Materials and Methods

### 2.1. Principle of Rotating Shaft Vibration Measurement

Rotating shaft vibration is one of the parameters that characterize the state of powerful generators. The character and magnitude of the rotating shaft vibration for each type of machine depend on their design features and operating conditions. These parameters should not exceed the specified norms defined by standards and regulatory documents [31,32,33,34,35,36,37,38,39].

Rotating shaft vibration is divided into relative and absolute radial shaft vibration. Typically, rotating shaft vibration in high-power generators is measured in the upper generator bearing, the lower generator bearing area, and the area of the turbine bearing. In most cases, in vertical hydro generators, the displacement value between the shaft and the sensor caused by relative radial shaft vibration is smaller than the maximum value of the absolute radial shaft vibration [40]. This makes it necessary to use several sensors at the same time to calculate the correct relative and absolute radial shaft vibration values and, in most cases, to use the values obtained from other sensors (temperature, shaft vibration, air gap clearance, body vibration, and other parameters) to correct the calculations values of the shaft vibration.

**Figure 1:**
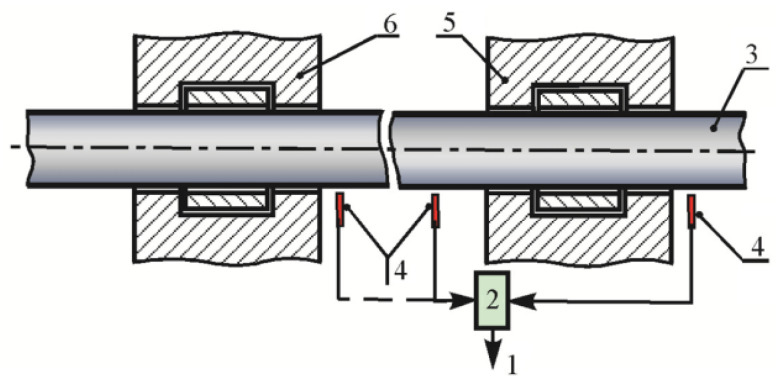
Rotating shaft vibration sensor mounted points: 1—fault diagnostic system with special processing tools, 2—data acquisition, 3—rotor shaft, 4—sensors, 5—housing, 6—bearing.

Feature sensor installation and value calculation of relative radial shaft vibration is a type of shaft vibration that is measured relative to the bearing body. Two non-contact sensors with an angle of 90° between them are annexed to the relevant part of the bearing body in the X and Y directions, as specified in ISO 10817 [37]. The scheme in Figure 1 shows the mounting point for a rotating shaft vibration sensor.

The sensors can be mounted either on the bearing housing or directly in the bearing guides. This arrangement of sensors allows the position of the shaft in the bearing gap to be determined. As rotating shafts, vibration sensors can be mounted on bearing housings identically using eddy current and capacitive sensors.

Eddy current sensors are mounted inside the bearing, where the magnetic and electric fields of the machine are not influenced in terms of their response function. Moreover, eddy current sensors used in the presence of oil film areas are not as sensitive as capacitive sensors. Capacitive sensors have the advantage of being installed on the outside; the performance of the sensors is not affected by the magnetic and electric fields of the machine [18,19].

**Figure 2:**
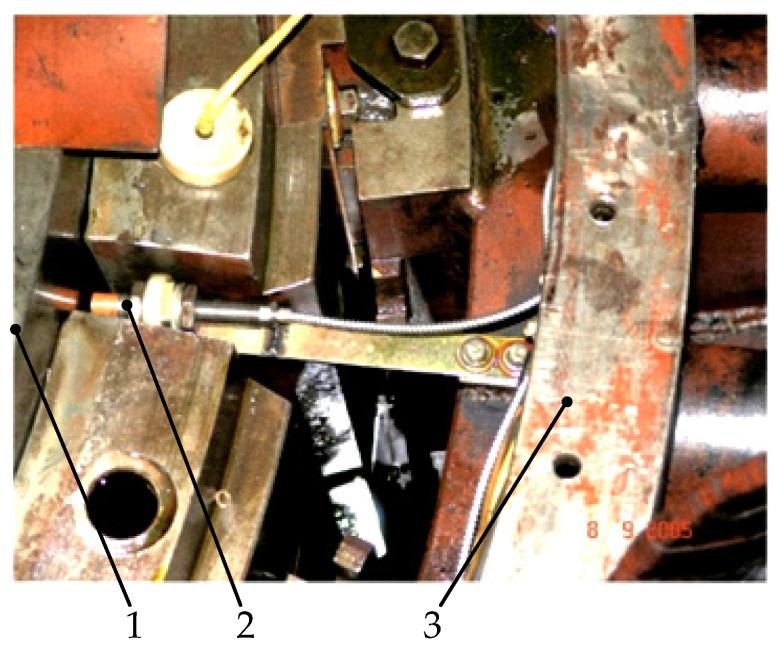
Installing sensors for rotating shaft vibration measurement: 1—shaft, 2—sensor, 3—housing.

Figure 2 shows an example of the placement on the cover of the rotating shaft vibration sensor PCS-200 ESB from VibroSystM Inc. (Longueuil, QC, Canada) [41]. The range of measurements of radial vibrations of bearings is approximately several hundred micrometers (microns). Therefore, to control the movement of the shaft using sensors in both the X-direction and the Y-direction installed next to the guide bearings, sensors with a measuring range of less than 2 mm are used [42].

To determine the magnitude of the absolute radial vibration, the vibration of the shaft is measured relative to the stationary part of the machine. In this case, the measuring sensor is closely connected to the foundation. Absolute and radial vibration measurements can be performed using the same configuration of non-contact XY sensors with additional elements.

Two methods are used for measuring the absolute vibration in power generator control systems. The use of different methods is related to the frequency of the rotation of the generators. At high speeds of the generator shaft, there may be vibrations on the shaft that need to be compensated. For such compensation, additional sensors are used. At low frequencies of rotation of the generator shaft, high-frequency vibrations are extremely rare, and their appearance is associated with defects and malfunctions of the generator. Consider the use of sensors for rotating shaft vibration measurement in each of the following methods.

**Figure 3:**
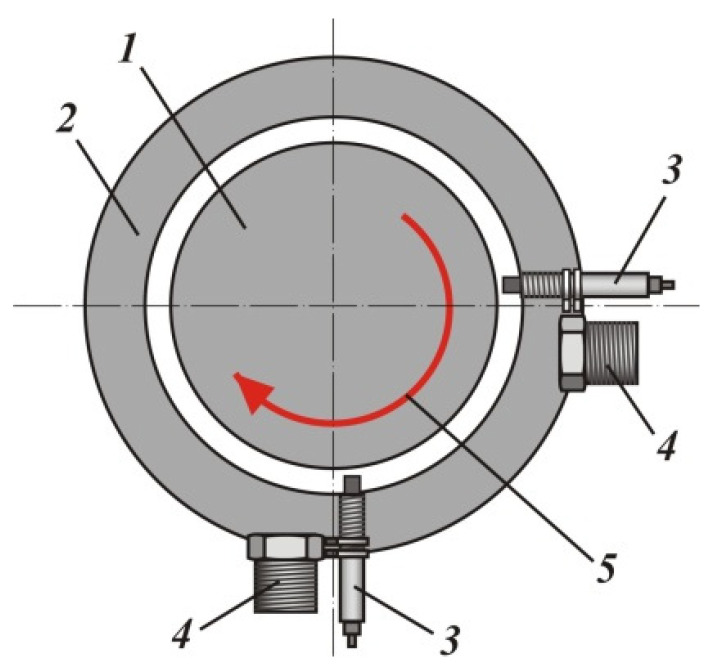
Contactless XY sensor installation scheme: 1—shaft of the hydraulic unit; 2—bearing housing; 3—non-contact sensor of relative radial vibration of the shaft; 4—vibration sensors of the bearing housing (accelerometer); 5—direction of rotation.

The first method used for controlling rotating shaft vibration hydro generators is to operate a system of contactless XY sensors simultaneously with a system of accelerometers [41,43]. Figure 3 shows the scheme of installing sensors for measuring rotating shaft vibration; 1 indicates the shaft of the hydraulic unit, 2 indicates the bearing housing, 3 indicates the non-contact sensor of relative radial vibration of the shaft, and 4 indicates vibration sensors of the bearing housing (accelerometer). The requirements for accelerometers are set by international standards [44,45].

Using double integration, the measured vibration acceleration is converted into vibration displacement and determines the absolute vibration for both the X and Y coordinates; these vibration displacements are vectors summed with the displacements measured by contactless sensors.

The CoDiS PMU Portable Monitoring Unit, developed by IRIS Power LP (Mississauga, ON, Canada), uses the described method. The system is used at HPP Jajce-1 (Jajce, Bosnia and Herzegovina) to measure the absolute vibration of the Francis Hydropower Unit with a capacity of 31 MW and a speed of 300 rpm [43].

The second method is used for measuring absolute vibration. For low-speed hydraulic units, the same configuration of XY sensors (Figure 3) is used, but the sensors are mounted on the wall of the generator using orthogonally oriented rigid brackets, which allows the measurement of the absolute vibration of the rotor relative to the fixed part of the structure.

**Figure 4:**
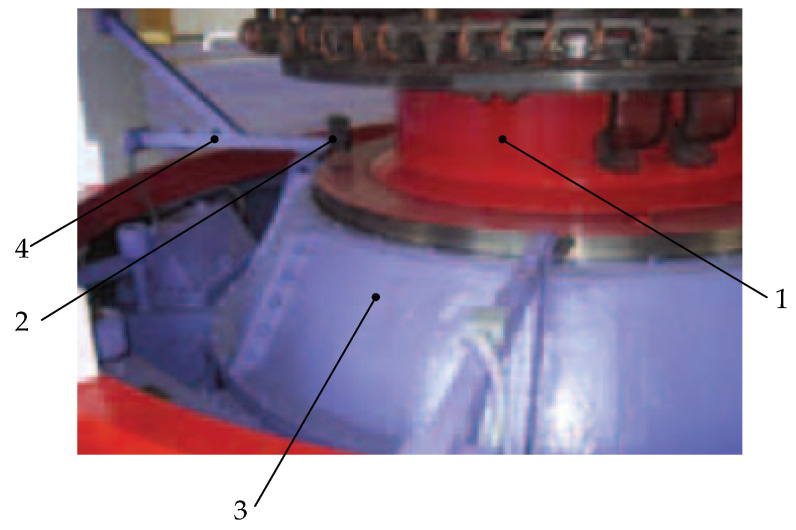
Installing absolute vibration sensors: 1—shaft, 2—sensor, 3—housing, 4—sensor mount.

Figure 4 shows an example of the installation of absolute vibration sensors on two rigid brackets near the guide bearing of the hydraulic unit, where the brackets themselves are connected to a fixed structure [42].

It is necessary to ensure reliable mechanical contact of each sensor for both relative and absolute vibration with the part on which it is installed. Installing the sensors on shelves or brackets allows vibration measurement to be achieved without distortion and vibration imposition from other elements of the generator structure. The natural frequency of mechanical vibrations of the shelf or bracket with the transducers should not coincide with the frequency of any component of the measured vibration. The vibration of any section of the surface of the rotating shaft for the hydraulic unit is characterized by the time change of the position of the central point of the shaft only in the area relative to a fixed point of the sensor electrode plate.

Accurate calculation of the amplitudes and phases of the harmonic components is necessary to build reliable characteristics of the change in the beating of the shaft over time. If we consider each sensor separately, we will have an incomplete picture of the state of the generator unit. To solve this problem, it is necessary to bring the harmonic components of all sensors mounted on the shaft to one point in time, i.e., to determine the initial phases of each harmonic or synchronize the operation of the sensors in time. This allows a clearer diagnosis of the state of the generator unit.

The spectral representation of the beating signal, namely its amplitude spectrum, can be used to analyze the vibration state of the hydraulic unit [37]. Various mathematical algorithms are used to obtain the spectral characteristics of the beat signal. A discrete Fourier transform is usually used to calculate the beating signal’s spectrum in implementing the algorithm to calculate the harmonic components’ amplitudes and initial phases [46].

### 2.2. Capacitive-Based Sensors with Concentric Electrodes for Rotating Shaft Vibration Measurement System

In modern, powerful monitoring and diagnostic systems, contactless capacitive sensors with different electrode geometries are used to measure shaft vibration [47]. In such sensors, one of the electrodes is a grounded shaft.

**Figure 5:**
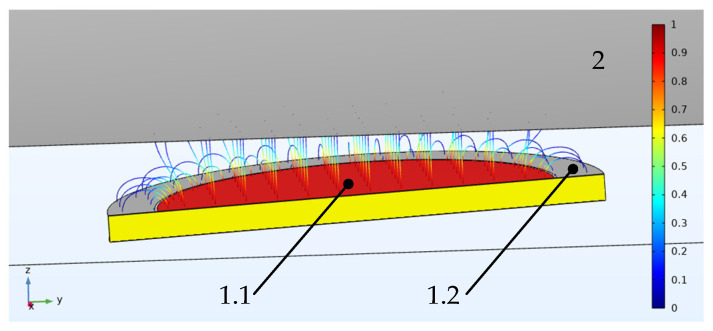
Rotating shaft vibration capacitive type 1 sensors with concentric electrodes: 1.1—potential electrode, 1.2—ground, 2—shaft.

Figure 5 shows the capacitive sensor (called a type 1 sensor below). This type of sensor is the most common for measuring rotating shafts’ vibration. In this sensor, the work capacitance value *C_i_* (between electrode 1.1 and grounded shaft 2) is defined as:

(1) $$C_{i}=\varepsilon_{0}\varepsilon_{r}\pi r^{2}/d_{i}+\delta (d_{i}/R)$$

where ε_0_ = 8.8542 × 10^−12^ F/m and is the dielectric constant of vacuum; ε*_r_* = 1.00056 and is the relative dielectric constant of air; *r* is the radius of the potential electrode 1.1; *R* is the radius of the shaft; *d* is the distance between the total plane of the electrodes and the grounded surface of the shaft 2; *δ*(*d*/*R*) is an additional component of the capacity due to the fringe effect.

The shaft radius (shown in Figure 5 as element 2) is much larger than the radius of the sensor. For this sensor, the calculation model assumes that the system surface of the sensor—the surface of the shaft—is plane-parallel. Taking into account the experimental studies carried out on models and full-scale samples described in previous works, the disadvantage of such a sensor is the significant error from the fringe effect, especially when *d*/*2r* > 0.1 [48,49,50].

**Figure 6:**
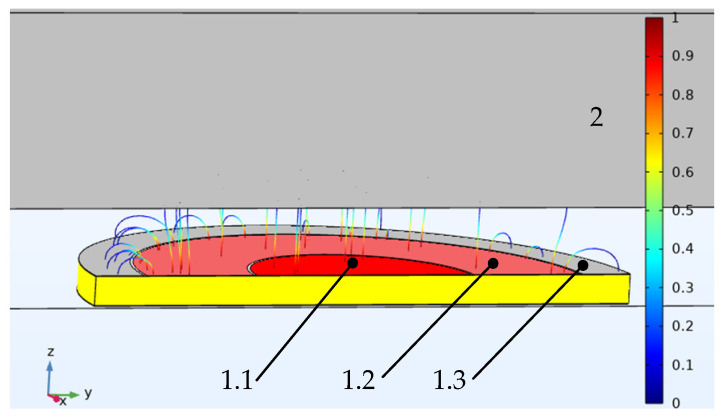
Rotating shaft vibration capacitive type 2 sensors with concentric electrodes: 1.1—potential electrode, 1.2—guard electrode, 1.3—ground, 2—shaft.

This is minimized by placing a protective electrode in the plane between the potential electrode and the grounded electrode, which is not galvanically connected to it. The addition of a concentric guard electrode to the structure results in a uniform electric field with parallel power lines for the potential electrode (Figure 5, shown as element 1.1) created in the working gap of the sensor (between the potential electrode and the ground plane), which eliminates the influence of the fringe effect on the measurement result. This type of sensor is conventionally referred to as a type 2 sensor with concentric electrodes. Figure 6 shows the type 2 sensor.

The sensor (Figure 6), which measures the distance d to grounded shaft 2, consists of potential electrode 1.1, with radius *R* and a protective electrode 1.3 width b, and grounded electrode 1.2, the size of which is determined for design reasons. The width *b* of the protective electrode depends on the ratio *d*/*R* and is calculated for a given error. Electrodes 1.1, 1.2, and 1.3 are separated by the dielectric interval *h*, which must have a minimum width for greater field uniformity. For the secondary measuring transducer (not shown in Figure 6), the sensor is connected by cable 6 with a double screen, and the central core of the cable is connected to potential electrode 3, the inner screen is connected to guard electrode 1.3, and the outer one is connected to grounded electrode 2.

The working capacity of the sensor is calculated by Equation (1). A strictly linear design characteristic characterizes sensors of this type, and they provide high accuracy and resolution.

## 3. Results

### 3.1. Determination of Ratios for Calculating the Optimal Geometric Dimensions of the Radius for the Potential Electrode of the Sensor with Concentric Electrodes

We used analytical calculations for the sensor with concentric electrodes to determine the optimal geometric dimensions, which were conducted in [51]. Using the mathematical relationship from [51] and Equation (1), the sensor response function was calculated as:

(2) $$C_{i}=\varepsilon_{0}\varepsilon \frac{\pi r^{2}}{d_{i}}+\delta (d_{i}/R)=\varepsilon_{0}\varepsilon \frac{\pi r^{2}}{d_{i}+R-\sqrt{R^{2}-x_{i}^{2}}}+\delta (d_{i}/R)$$

where *x* is the distance between the surface of shaft 2 and the plane of the surface of the sensor’s electrode, which has an infinitesimal width and diameter equal to the distance *b* (calculated considering the shaft diameter).

Analyzing Equation (2) and the mathematical relationship from [51] the response function, and measurement errors were influence values of the radius of the potential and guard electrodes, *r* and *b*.

**Figure 7:**
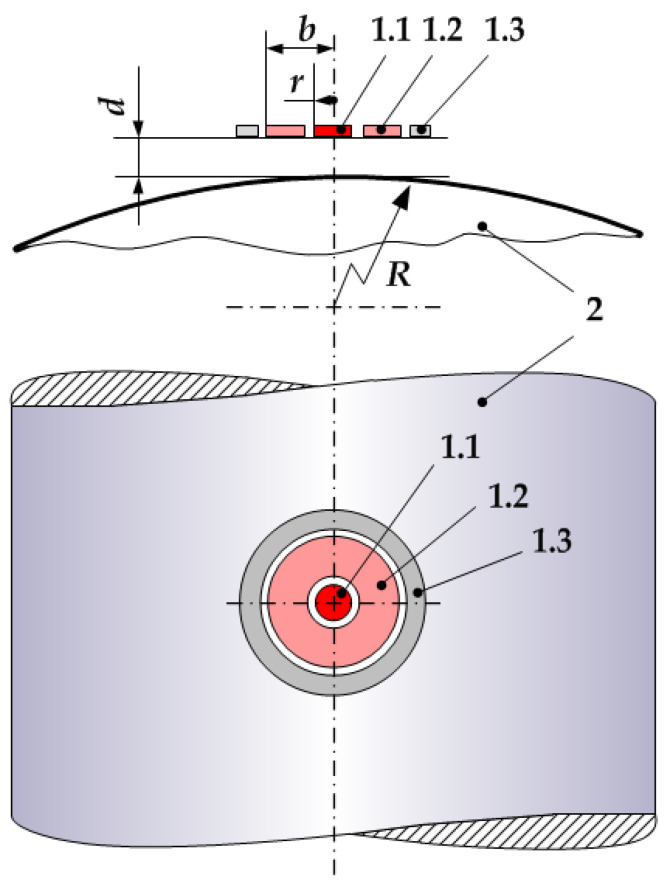
The scheme of calculating the capacitive sensor electrode sizes for the rotating shaft vibration measurement system: 1.1—potential electrode, 1.2—guard electrode, 1.3—ground, 2—shaft.

Figure 7 shows a diagram for calculating the optimal dimensions *r* and *b*, considering the radius of the shaft *R* and the range of distance measurement *d*. The choice of optimal parameters allowed the minimization of errors in measuring the vibration parameters of rotating shafts.

For the sensor with the configuration of the electrodes shown in Figure 7 [51], the value of the distance *d*, measured with it, was calculated as:

(3) $$d_{i}=k\times C_{i}^{-1}$$

where *C_i_* is the electrical capacity of the capacitor formed by the plane of electrode 1.1 and the surface of shaft 2; *k* is the proportionality coefficient.

The analysis of Formulas (2) and (3) and the mathematical relationship from [51] show that at constants *r* and *R*, the error of the distance measurement due to the curvature of the shaft increases with a decreasing distance *d*. This adds requirements for selecting the sensor’s initial (minimum) distance to the measuring object (shaft). It should be chosen on the basis of the specified allowable measurement error. On the other hand, the same error increases with increasing *r* and decreasing *R*.

Usually, the sensor is installed on the finished specific machine, and because of this, the radius *R* is known in advance. Therefore, to ensure the specified accuracy, a sensor was chosen with a certain size of the potential electrode with radius *r*.

How the curvature of the shaft changed the error by (with distance to the shaft surface *d*) was defined for several values of the radius *r* of the potential electrode of the sensor. In the calculation, it is convenient to use the coefficient *A* as a variable value, which is determined by the ratio *A = R*/*r*, i.e., *R* = *A* × *r*. Therefore, in this case, Equation (3) and the mathematical relationship from [51] for capacitive *C_i_* was used, calculated as:

(4) $$\begin{array}{l} C_{i}=4\varepsilon_{0}\varepsilon \{\frac{\pi }{2}\left(d_{i}+R\right)\left(\frac{\sqrt{d_{i}^{2}+r^{2}+2d_{i}R}}{\sqrt{d_{i}}\sqrt{d_{i}+2R}}-1\right)-\\ -\left(\begin{array}{l} -d_{i}R^{2}\left(d_{i}+2R\right)\mathrm{EllipticE}\left[K_{1},K_{2}\right]-\left(d_{i}{}^{2}+r^{2}+2d_{i}R\right)\times \\ \times \left(\begin{array}{l} d_{i}\left(d_{i}+2R\right)\mathrm{EllipticF}\left[K_{1},K_{2}\right]-\\ -{\left(d_{i}+R\right)}^{2}\mathrm{EllipticPi}\left[-\frac{r^{2}}{d_{i}{}^{2}+2d_{i}R},K_{1},K_{2}\right] \end{array}\right) \end{array}\right)\times {\left(K_{3}\right)}^{-1}+\\ +\left(\begin{array}{l} -d_{i}K_{1}^{2}\left(d_{i}+2R\right)\mathrm{EllipticE}\left[i\mathrm{ArcSinh}\left[0\right],K_{2}\right]-\left(d_{i}{}^{2}+r^{2}+2d_{i}R\right)\times \\ \times \left(\begin{array}{l} d_{i}\left(d_{i}+2R\right)\mathrm{EllipticF}\left[i\mathrm{ArcSinh}\left[0\right],K_{2}\right]-\\ {\left(d_{i}+A-r\right)}^{2}\mathrm{EllipticPi}\left[-\frac{r^{2}}{d_{i}{}^{2}+2d_{i}R},i\mathrm{ArcSinh}\left[0\right],K_{2}\right] \end{array}\right) \end{array}\right)\times {\left(K_{3}\right)}^{-1}\} \end{array}$$

where $K_{1}=i\mathrm{ArcSinh}\left[\sqrt{-1/r^{2}}\times r\right]$, $K_{2}=r^{2}/R^{2}$, $K_{3}=d_{i}R\left(d+2K_{1}\right)$, EllipticF() is a function of the elliptic integral of the first kind, EllipticPi() is a function of the complete elliptic integral of the third kind, EllipticE()—a function of the complete elliptic integral, and ArcSinh() is a function of the inverse hyperbolic sine.

To determine the relative error *δ_p_* of the distance to the flat surface and the cylindrical surface of the shaft due to the radius of the potential electrode, the result of the determined distance to the shaft surface obtained by Equation (4) was compared with the value of the distance to the flat surface calculated using the plane-parallel capacitor Equation. The capacitance *C*_0_ of a plane-parallel capacitor with a round potential electrode having a radius *r* and a gap between the electrodes equal to *d* was determined by the next Equation [52,53]:

(5) $$C_{0}^{}=\varepsilon_{0}\varepsilon \frac{\pi r^{2}}{d}$$

The results obtained for one type of sensor with a round potential electrode with radius *r* were compared. The relative difference *δ_p_* in determining the distance to the flat surface and the cylindrical surface of the shaft in percent was calculated as:

(6) $$\delta_{p}=\left(C_{X}^{-1}-C_{0}^{-1}\right)/C_{0}^{-1}\cdot 100\%$$

**Figure 8:**
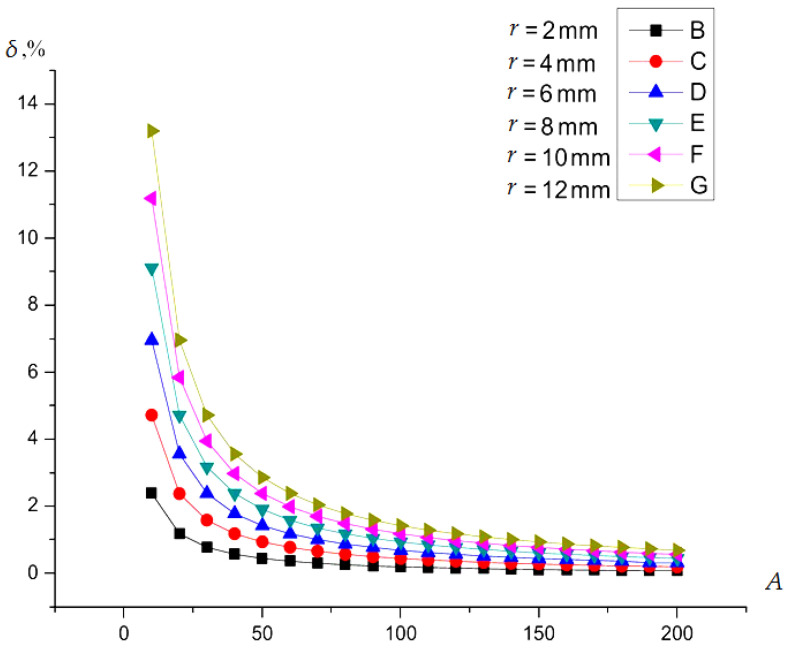
The error of measuring the distance to the cylindrical surface (shaft surface), depending on the ratio *A* = *R/r*.

Figure 8 shows the dependence of *δ_p_ = f*(*A*) for a number of sensors with different radiuses of the potential electrode *r*. Using the curves shown in Figure 8 and a choice the values *δ i* *R* can determine the value of *r*.

### 3.2. Determination of Ratios for Calculating the Optimal Geometric Dimensions of the Radius for the Guard Electrode of the Sensor

For a plane-parallel capacitor with the round active electrode, the protective electrode’s size was calculated in the 1950s and 1960s in a significant number of works, most of which were devoted to non-destructive testing. Different approaches were used in the calculation, which allowed the estimation of the deviation of the capacitance value, due to the final width of the protective electrode, from the capacitance of an ideal capacitor. For the case with the grounded surface as one of the sensor’s electrodes, calculating the electrical capacitance of a plane-parallel capacitor using Green’s functions is the most convenient for practical use and the most accurate [54].

To determinate the capacitance of a capacitor in which one of the electrodes instead of an infinite plane is a grounded cylindrical surface of the shaft, we used a method based on Green’s functions.

A plane-parallel capacitor with a round active electrode and a protective electrode located around it will be perfectly shielded when the radius of the protective electrode is infinitely large (*b*→∞) and the second electrode of the capacitor is an infinite ground plane. Such a capacitor has no fringe effect, i.e., there is a perfect plane-parallel electric field. The capacity of such a capacitor is determined by the known Equation (5). If the value of *b* is finite, the capacity *C* will decrease and be determined [54] as:

(7) $$C=C_{0}(1-\gamma )$$

where *γ* is the value of the error by fringe effect.

According to Equations (5) and (7) and the geometric dimensions of the sensor *r*, *b*, and *d*, the precise values of the error *γ* were calculated as [54]:

(8) $$\gamma =-\frac{4}{\pi }\frac{d}{r}\sum_{n=1}^{\infty }\left({\left(-1\right)}^{n}\frac{1}{n}I_{1}\left(n\pi \frac{r}{d}\right)/I_{0}\left(n\pi \frac{b}{d}\right)\right),$$

where *I*_0_, *I*_1_ are the transformed Bessel functions depending on the *Z* coordinate (it passes through the center of the active electrode perpendicular to its plane).

Under the condition that *d* << *r* << *b*, *d* << (*b* − *r*), *z* → ∞, the following approximate ratios are true:

(9) $$I_{0}(z)\approx \frac{e^{z}}{\sqrt{2\pi z}},\;I_{1}(z)\approx \frac{e^{z}}{\sqrt{2\pi z}},$$

Using Equations (7) and (8), the approximate value of the error *γ* can be determined as:

(10) $$\gamma \approx \frac{4}{\pi }\left(\frac{d}{r}\right)\sqrt{\frac{b}{r}}\times e^{-\pi \cdot \frac{b-r}{d}}$$

**Figure 9:**
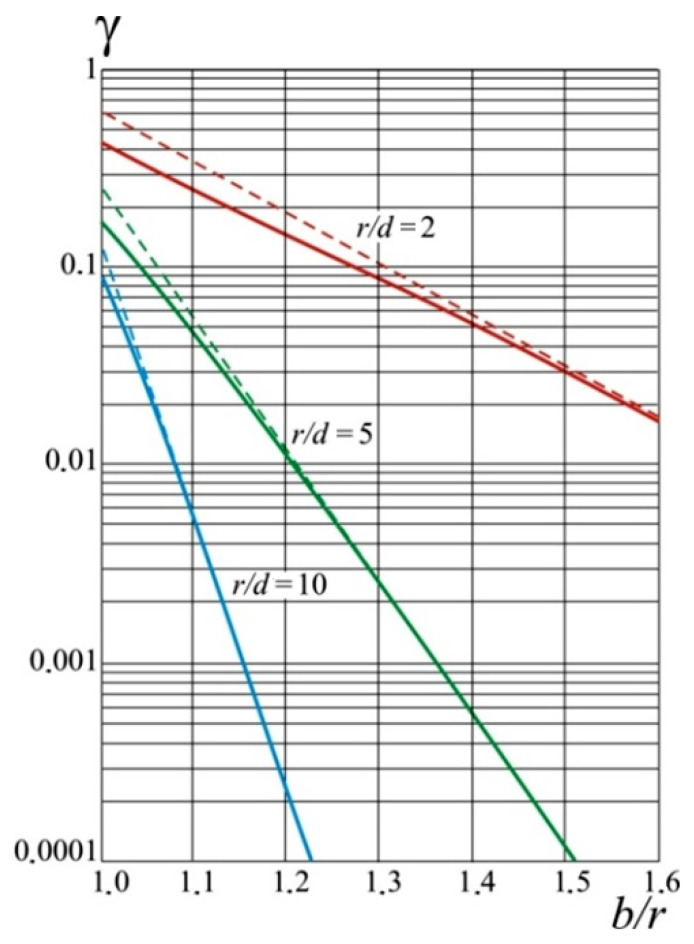
Dependence of error *δ* = *f*(*b*/*d*) from the value of the radius of the guard electrode.

Figure 9 shows a curve family for different values of the ratio *r*/*d* for determining *δ = f*(*b*/*d*). The solid curve shows the exact values calculated using Equation (9) and the approximate values (dotted curve) obtained by Equation (10).

The curves in Figure 9 show that *δ* decreases faster than the *r*/*d* ratio. For a specific value of *r/d*, the error *γ* becomes smaller with an increasing *b*/*r* ratio. The choice of the values of *γ*, *r*, and *d* and the use of the curves in Figure 9 can determine the radius of the guard electrode *b*.

For high-power electric machines, the average dispersion of the radial beating range of the shafts was 2 to 4 mm [37,40]. If we take the value of the minimum gap to shaft surface for the capacitive beating sensor as equal to *d*_min_ = 1 mm, then, taking into account the amplitude of the beating, the maximum clearance will be *d*_max_ = 5 mm. The results show that the maximum error corresponds to the gap between the sensor electrode plane and the shaft surface.

It is convenient for practical engineering calculations to have graphs and analytical dependences of the error *γ* not on the radius of the protective electrode *b* but on its width *c* at the maximum clearance *d* = *d*_max_ or the clearance dx set manually by the engineer. Given that the width of the guard electrode is defined as *c = b − r*, Equation (10) is written as:

(11) $$\gamma \approx \frac{4}{\pi }\sqrt{1+\frac{c}{r}}\times e^{-\frac{\pi \cdot c}{d}},$$

**Figure 10:**
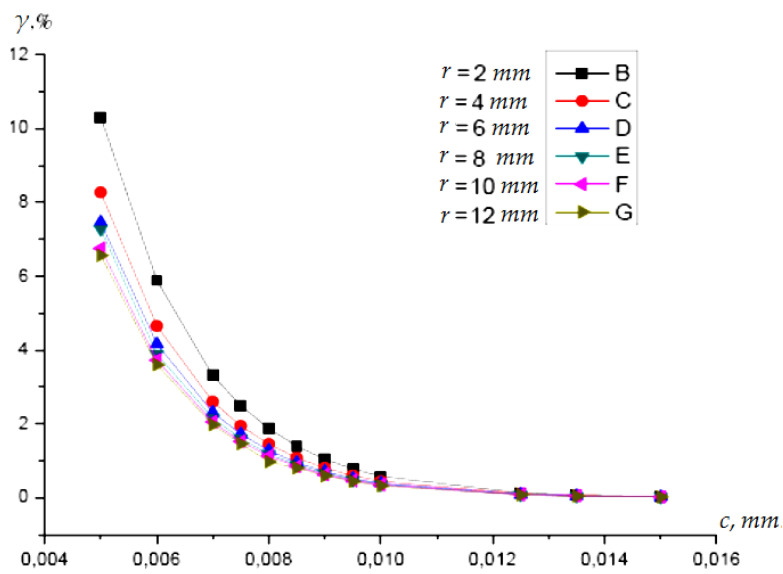
Dependence of the error *γ = f*(*c*) from the width of the guard electrode (*d* = 5 mm).

Figure 10 shows the curve family *γ* = *f*(*c*) for different values of the active electrode radius *r* and the maximum gap to shaft surface *d_max_* = 5 mm.

Using Formula (11) and the dependences shown in Figure 9 allows the width *c* of the security electrode to be determined at a given error value *γ.*

### 3.3. Definition of the Response Function Use Computer Simulation

Using the relationships obtained in the previous section, the response function was characterized using computer simulation. The computer model implementation environment was COMSOL Multiphysics. The optimal dimensions of the sensor electrodes were calculated using the ratios shown in Figure 8, Figure 9 and Figure 10. The resulting design dimensions of the sensor had the following values: *r*_1_ = 4 mm, the radius of the active electrode of the type 1 sensor; *r*_2_ = 4 mm, the radius of the active electrode of type 2 sensor; *b*_2_ = 8 mm, radius of guard electrode of type 2 sensor; 1 mm width of the ground electrode of the sensor; material FR-4 with a dielectric constant 4l and *h* = 0.1 mm, the value of air gap between the electrodes of type1 and type2 Dimension values were calculated using Figure 9 and Figure 10, considering that the maximum allowable external diameter of the sensor could be no more than 20 mm and that the shaft diameter value was *R* = 800 mm (capsular Hydro generator SGK 538/160–70M).

**Figure 11:**
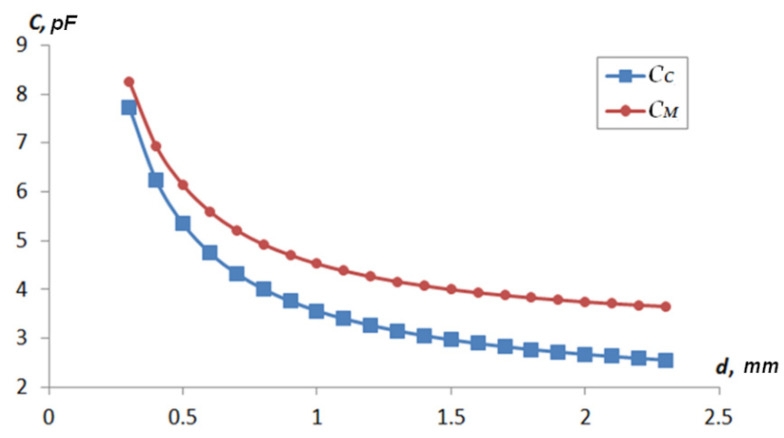
Type 1 sensor response function depending on the distance to the shaft surface.

**Figure 12:**
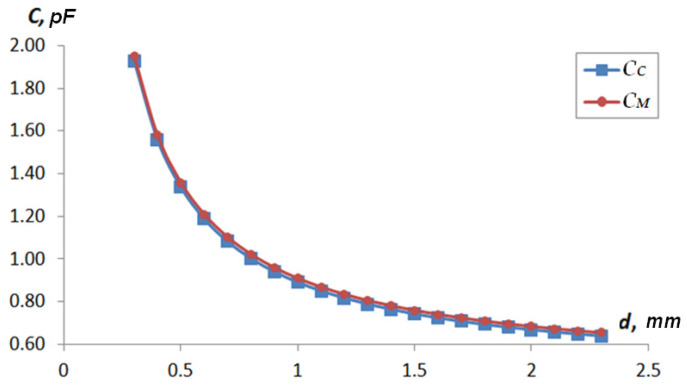
Type 2 sensor response function depending on the distance to the shaft surface.

Table 1 shows the results of determining the value of the capacitance *C_x_*(*d_x_*) = *f*(*d_x_*) for each type of sensor: *C_C_* represents the value of the capacitance determined by analytical methods and *C_M_* represents the capacity values determined by computer simulation. The response functions were calculated using two methods to determine the possibility of using a 3D-FEM model and analytical relationships. Figure 11 and Figure 12 show the graphs of *C_C_ = f*(*d*) and *C_M_* = *f*(*d*).

**Figure 13:**
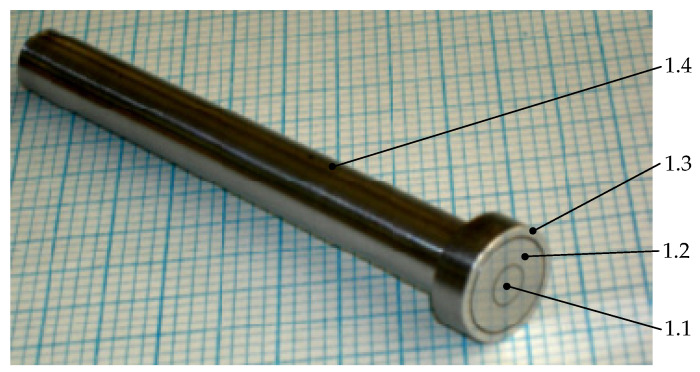
Prototype of type 2 sensor: 1.1—potential electrode, 1.2—guard electrode, 1.3—ground, 1.4—sensor body.

The natural sensor was implemented to check the validity of the analytical calculations and simulation results. To implement the sensor, the sizes of the electrodes used for analytical calculations and models were used. Figure 13 shows the type 2 sensor test model. 

**Figure 14:**
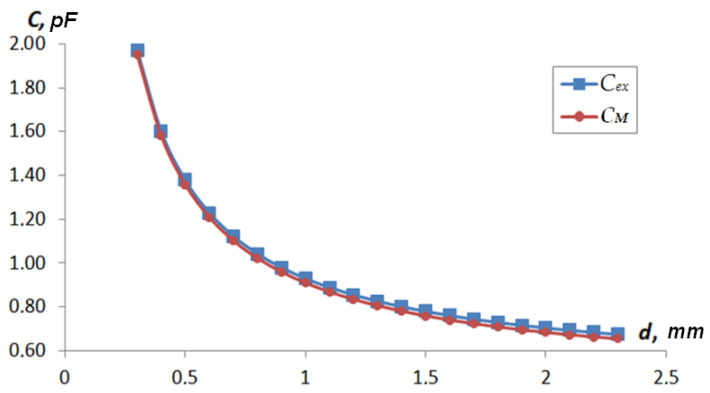
Comparison of experimental and model data for type 2 sensor.

Figure 14 shows the result of the experimental study of the type 2 sensor test model. In Figure 14 *C_ex_* experimental study result. The difference in the results between the FEM model and the real sample was 0.01 pF on average in the measuring range. The nature of the additional capacitance was additive and was related to the capacitance of the connecting lines between the sensor and the measuring transducer. A comparison of the results showed almost complete agreement between the results of the experiment and the simulation.

### 3.4. Determination of Error by the Fringe Effect

The results obtained in Table 1 show a significant capacity *Cp* by the fringe effect. The value of the error caused by the influence fringe effect on the value of the capacitance *Cp* was determined. To do this, we used analytically obtained data and simulation data.

**Figure 15:**
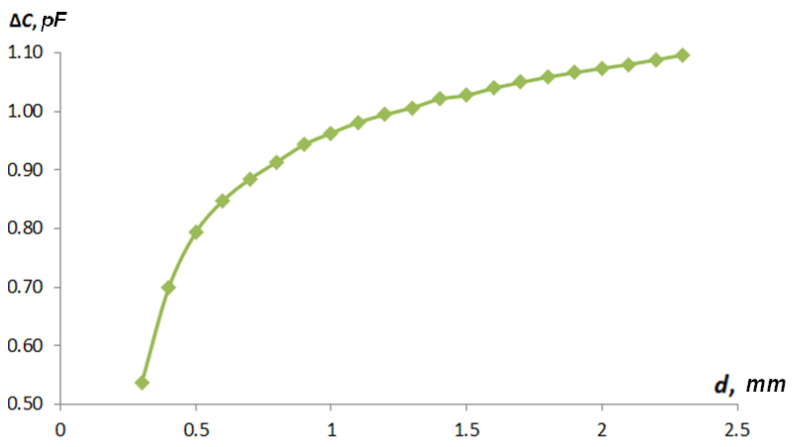
Graphs of the influence of the fringe effect on the total capacity of the distance to the grounded control object for the type 1 sensor.

**Figure 16:**
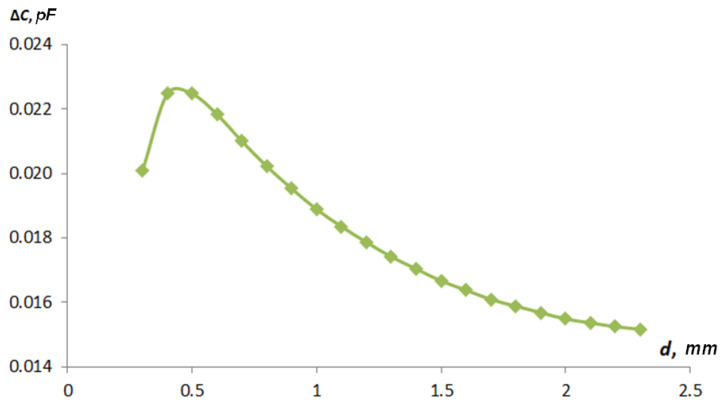
Graphs of the influence of the fringe effect on the total capacity of the distance to the grounded control object for the type 2 sensor.

Figure 15 shows a graph of the influence of the fringe effect on the total capacity from the distance to the grounded control object for sensor type 1, and Figure 16 shows that for type 2.

As can be seen in obtained curves in Figure 15 and Figure 16, it can be concluded that the discrepancy between the values of the capacitance determined by the analytical method and the means of computer modeling is caused by the presence of parasitic capacitance, which occurs because of the curvature of electric field lines, i.e., the fringe effect. The type 1 sensor was more affected by the fringe effect than the type 2 sensor. The curves in Figure 15 have a complex character. First, there is a rise, which is due to the fact that at very small gaps, the parasitic capacitance is small, and with an increase in the gap, the parasitic capacitance is formed between the active electrode and the textolite substrate through a grounded shaft. As the sensor moves away from the shaft surface, this connection drops. Such a discovered effect will require further careful studies on FEM models for the correct construction of an algorithm of full-scale tests that are able to confirm the effect obtained.

## 4. Conclusions

This paper proposed the optimal design of a capacitive sensor with concentric electrodes for measuring rotating shaft vibration. The sensor’s principle of operation was based on measuring the value of the capacity from the distance to a metal surface, which allows increasing reliability of operation of powerful turbine and hydro generators. The study results of the characteristics of capacitive rotating shaft vibration sensors using simulations in COMSOL Multiphysics were obtained. Using the computer simulation methods, the response functions of capacitive sensors were determined depending on the distance between the plane of the sensor electrodes and the grounded surface of the test object (shaft surface) for sensors with a variety of electrodes. Tabular and graphical results of determining the response functions of sensors were presented. The results of the simulation and analytical mathematical models showed that the models created based on analytical mathematical models can be used to predict the response functions of sensors. As a result of the calculations, it was obtained that for the type 1 sensor, the value of the largest discrepancy was more than 1 pF at the greatest distance to the grounded surface. For the type 2 sensor, the parasitic capacitance value was 0.02 pF and decreased with increasing distance to the grounded control object. On the basis of the obtained results of the analytical calculation and modeling of response functions, the non-linearity error was calculated. It was the same for the type 1 and 2 sensors. The average error value for the type 1 sensor was 0.01 pF, and that for the type 2 sensor was 0.04 pF. The comparison of the results of the analytical calculations, modeling, and field studies shows the possibility of using the proposed relationships for designing sensors.

## Figures and Tables

**Table 1:** The calculations result in the difference between response function capacities determined analytically and using computer simulations.

	Type1		Type2	
*d*, mm	*C_C_*, pF	*C_M_*, pF	*C_C_*, pF	*C_M_*, pF
0.3	7.72	8.26	1.48	1.95
0.4	6.23	6.93	1.11	1.58
0.5	5.34	6.14	0.89	1.36
0.6	4.75	5.60	0.74	1.21
0.7	4.33	5.21	0.64	1.10
0.8	4.01	4.92	0.56	1.02
0.9	3.76	4.70	0.49	0.96
1	3.56	4.53	0.45	0.91
1.1	3.40	4.38	0.40	0.87
1.2	3.27	4.26	0.37	0.83
1.3	3.15	4.16	0.34	0.81
1.4	3.05	4.07	0.32	0.78
1.5	2.97	4.00	0.30	0.76
1.6	2.89	3.93	0.28	0.74
1.7	2.83	3.88	0.26	0.72
1.8	2.77	3.83	0.25	0.71
1.9	2.72	3.79	0.23	0.70
2	2.67	3.75	0.22	0.68
2.1	2.63	3.71	0.21	0.67
2.2	2.59	3.68	0.20	0.66
2.3	2.56	3.65	0.19	0.65

## Data Availability

The data presented in this study are available upon request from the corresponding author.
